# Type A and Type B Alcoholism: Applicability Across Subpopulations and Treatment Settings

**Published:** 1996

**Authors:** Samuel A. Ball

**Affiliations:** Samuel A. Ball, Ph.D., is an assistant professor at the Yale University School of Medicine, Department of Psychiatry, Division of Substance Abuse, New Haven, Connecticut

**Keywords:** AOD dependence, disorder classification, patient assessment, disease course, racial differences, risk factors, high risk group, treatment outcome, applied research, gender differences, patient-treatment matching

## Abstract

Babor’s classification of alcoholism uses multiple characteristics to assign subjects to two categories, called type A and type B. Type B alcoholism appears to be consistently more severe than type A. Research findings are reviewed that support the usefulness of Babor’s typology for different drugs of abuse, clinical settings, gender, and race.

Researchers have long attempted to categorize alcoholics based on various defining characteristics, or dimensions. These attempts reflect the understanding that alcoholism is not a single disease process but a complex biopsychosocial disorder with many different causes, complicating factors, courses (i.e., expression and progression of symptoms), and outcomes. Although some alcoholism typologies have been based on a single dimension (e.g., early versus late onset), typologies involving multiple dimensions may characterize subjects more accurately while predicting a broader range of outcomes (Babor et al. 1988, 1992*a*).

Typological studies often use a computerized statistical technique called cluster analysis to group subjects within a population based on multiple dimensions. Dimensions relevant to alcohol and other drug (AOD) disorders^1^ include factors that precede the disorder (e.g., family history, personality, childhood behavior problems, and age of onset of AOD problems), severity of symptoms (e.g., amount and frequency of AOD use), and adverse medical and psychosocial consequences of AOD use (Babor et al. 1992*b*).

Evidence from both treatment (Babor et al. 1992*b*) and adoption (Cloninger 1987) studies suggests that all subjects in any sample of alcoholics can be assigned to one of two types that differ consistently in multiple dimensions (see table 1). This article explores Babor and colleagues’ two-type model.

## Type A and Type B Alcoholism

The typologies introduced by Babor and colleagues (1992*b*) and Cloninger (1987) are similar despite minor differences.^2^ Both Babor’s type A and Cloninger’s type I alcoholism are characterized by a later age of alcoholism onset, weaker family history (i.e., fewer first-degree relatives who are alcoholics), less severe dependence, fewer symptoms of co-occurring psychiatric disorders, and less psychosocial impairment (i.e., negative familial, social, legal, or occupational consequences of drinking). Conversely, Babor’s type B and Cloninger’s type II refer to a more severe alcoholism, characterized by earlier onset; stronger family history; more impulsive behavior and childhood conduct problems; more severe dependence; multiple drug abuse; and co-occurring psychiatric disorders, especially antisocial personality disorder (ASPD).^3^

The clinical usefulness of any typology lies, in part, in its ability to help explain the different causes, courses, prognoses, and outcomes for a disorder. In addition, a typology for AOD abusers should apply to a wide range of drugs; treatment types; and demographic dimensions, such as age, gender, socioeconomic status, and race. Based on this premise, the following sections will examine type A and type B alcoholism.

## Applicability to Drugs Other Than Alcohol

Many AOD abusers use more than one drug. Therefore, an important issue for typological research is whether the dimensions for alcoholism types apply to other drugs as well. Researchers have found elevated rates of ASPD, depressive disorders, anxiety disorders, and multiple drug abuse among alcohol, cocaine, and opiate users (Rounsaville et al. 1982, 1991) and their first-degree relatives^4^ (Mirin et al. 1991). In addition, the symptoms used to diagnose alcohol dependence seem to apply to cocaine and opiate dependence (Kosten et al. 1987). Thus, abusers of a range of drugs often share similar risk factors, symptoms, and consequences of AOD use. Until recently, however, no study had examined whether these dimensions clustered into two groups similar to the type A and type B observed in alcoholism.

Ball and colleagues (1995) assessed 399 cocaine abusers based on dimensions (see table 2) similar to those used in Babor’s alcoholism typology study (Babor et al. 1992*b*). Cluster analysis revealed two well-defined types analogous to Babor’s type A and type B. Among the cocaine abusers, 33 percent were type A and 67 percent were type B. Compared with type A, the type B subjects exhibited greater evidence of risk factors preceding their disorder (e.g., childhood behavior problems and family history of AOD abuse), more severe AOD abuse, more psychological and social problems resulting from the disorder, and more coexisting psychiatric problems (e.g., ASPD). Further analyses indicated that type B subjects also had greater histories of aggression, criminality, violence, depression, suicide attempts, and treatment for either AOD abuse or psychiatric disorders. These subjects also exhibited greater quantity, frequency, duration, severity, and adverse effects of cocaine abuse and had an earlier age of onset for both alcohol abuse and ASPD compared with type A subjects. These results were generally consistent with Babor and colleagues’ alcoholism typology (1992*b*), suggesting broad applicability for this typological approach to AOD disorders.

Subsequent study confirmed that the distinction between type A and type B is also valid for opiate, cocaine, and marijuana abusers (Feingold et al. in press). For each drug, more subjects were assigned to the less severe type A group based on various AOD abuse and psychiatric measures administered at the initial interview and again at a 6-month followup. The only inconsistency was that age of onset and family history did not differ considerably between type A and type B, possibly because these dimensions were measured differently in this study than in other studies.

## Applicability to Subpopulations

### Gender

Several typologies emphasize gender as an important dimension in alcoholism. For example, Cloninger’s (1987) type II is essentially limited to men. Of the four types of alcoholism proposed by Zucker (1987), one type, associated with antisocial behavior, predominantly occurs in men, whereas another type, associated with anxiety and depression, appears to be more prevalent in women.^5^

**Figure:**
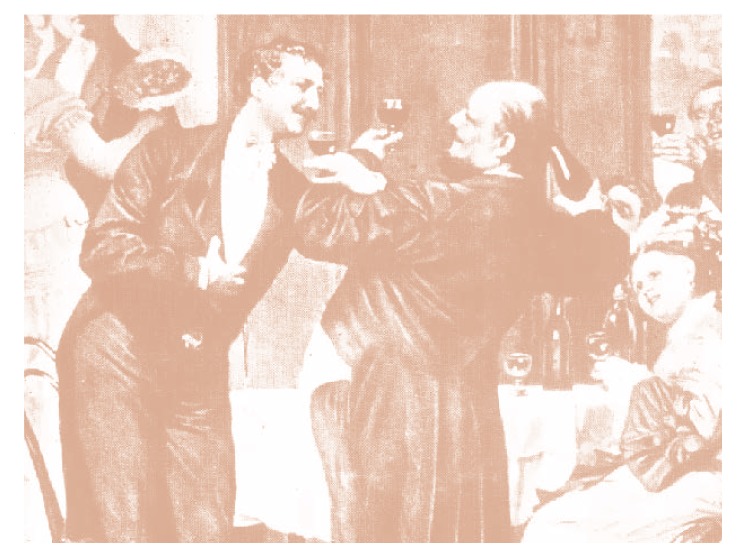
Type I/type A alcoholism illustrated in “Upper Class Social Gathering.” Original artwork for “Temperance Tales and the Alcoholic,” 1979. Reproduced with permission from the *Journal of Studies on Alcohol*. © Alcohol Research Documentation, Inc., Rutgers University Center of Alcohol Studies.

In Babor and colleagues’ alcoholism study (Babor et al. 1992*b*), men were equally classified as type A or type B, but women were more often classified as type A (62 percent). Interestingly, although type A and type B men differed from each other on all dimensions, type A and type B women did not differ on measures related to negative mood states (i.e., use of tranquilizers or consumption of alcohol to relieve withdrawal and psychological distress). In another study, a similar relationship was found between gender and typology in cocaine abusers (Ball et al. 1995). Although more cocaine abusers were assigned to type A than to type B, this difference was greater for women (79 percent type A) than for men (61 percent type A). Type A and type B men differed from each other on all dimensions, whereas type A and type B women did not differ from each other on measures related to family history of AOD abuse, lifetime psychiatric diagnoses, and severity of medical and legal problems.

Brown and colleagues (1994) also noted that more women (95.6 percent) than men (73.0 percent) were classified as type A. In contrast, a nationwide sample of alcoholics (Schuckit et al. 1995) found that proportionately more women (36 percent) than men (15 percent) were categorized as the more severe type B. Finally, Feingold and colleagues (in press) found no gender differences among a sample of AOD abusers. The reason for these discrepancies is unknown.

### Race

The usefulness of a given typology dimension may vary among different subpopulations. For example, variation in personality traits may be a key dimension for subjects from one racial group, whereas variation in drinking patterns may be more important for a different racial group. The relatively low numbers of minority subjects in several studies of type A and type B alcoholism (Babor et al. 1992*a*,*b*; Brown et al. 1994; Litt et al. 1992) provided no opportunity for researchers to determine the applicability of a two-type model or its dimensions across races. Although Schuckit and colleagues (1995) studied an ethnically diverse national sample of alcoholics, the researchers did not examine differences in the racial composition of types.

In a study of cocaine abusers, Ball and colleagues (1995) found that the type A-type B distinction was meaningful for both blacks and whites and that the dimensions were consistently more different for blacks than for whites. However, blacks were more commonly categorized as type A (74 percent) than were whites (63 percent). Feingold and colleagues (in press) found the same race difference in the typology for both cocaine and opiate abusers.

### Implications for Causality

The causes of AOD abuse are complex, involving the interaction of environmental factors and genetic predisposition. The two-type model seems to be fairly consistent across drug of choice, gender, and race. Although women and blacks are more commonly categorized as type A compared with men and whites, a significant number of women and blacks exhibit the kinds of risk factors, severity, impairment, and antisocial behavior previously thought to be more related to alcoholism among whites and men. If AOD abuse is more environmentally influenced among type A than type B, one might speculate that women and blacks may be more susceptible to developing AOD abuse for largely environmental reasons. Such information might help support the need for prevention programs targeted to specific populations. Future research should determine if this typology is valid for other ethnic minority groups in the United States, such as Hispanic, Asian, and American Indian populations, as well as for other cultures and countries.

## Applicability to Different Clinical Situations

The fact that “types” can be created statistically does not ensure that they have practical significance. To be clinically useful, a typology should facilitate treatment placement and planning decisions for a wide range of AOD abusers.

For any given disorder, one can generally assume that hospitalized patients (i.e., inpatients) are more severely ill than nonhospitalized patients (i.e., outpatients) and that patients in either treatment setting are more severely ill than people with the disorder who have not sought treatment (i.e., a community sample). Babor’s initial typology research (Babor et al. 1988, 1992*b*) focused on inpatient alcoholics and found roughly equal numbers of patients classified as type A and type B. Litt and colleagues (1992) found a similar 50:50 split among outpatient alcoholics; however, the typology dimensions were measured during inpatient hospitalization. Subsequent work by Brown and colleagues (1994) found that the majority of alcoholic outpatients were classified as the less severe type A (78 percent). Schuckit and colleagues (1995) found the A–B typology to be meaningful in samples of inpatient, outpatient, and community alcohol abusers, although the researchers did not determine the proportions of type A versus type B subjects in these samples.

Ball and colleagues (1995) applied the A–B typology to cocaine abusers in the three settings. The inpatient cocaine abusers included approximately equal numbers of type A and type B, as previously found with inpatient alcoholics (Babor et al. 1992*b*). Subjects in the outpatient and community samples were predominantly type A (75 percent), as previously found with outpatient alcoholics (Brown et al. 1994). Type A and type B inpatients differed from each other on all dimensions except recent and lifetime cocaine use. Type A and type B outpatients did not differ from each other on family history, personality type, depression, and recent and lifetime cocaine use. Type A and type B community subjects did not differ on alcohol dependence, age of onset, and lifetime psychiatric diagnoses. The differences between the three samples were not accounted for solely by differences in severity of drug dependence, because both the inpatients and outpatients were more severe cocaine abusers than were subjects who did not seek treatment.

Feingold and colleagues (in press) extended these findings in a sample of 521 abusers of opiates, cocaine, marijuana, and alcohol. The outpatient sample included general psychiatric patients as well as AOD abusers. Approximately 50 percent of the patients in drug treatment were categorized as type B, compared with 22 percent of the general psychiatric patients and only 5 percent of the community subjects. The researchers consistently observed these differences regardless of the drug used.

## Treatment Outcome and Selection

Babor and colleagues (1992*b*) found that type B alcoholics exhibited more severe AOD abuse, social problems, and psychological distress than type A alcoholics 12 months after initial assessment. Type B subjects relapsed faster and more often and needed additional treatment. Similarly, Ball and colleagues (1995) found that type B cocaine abusers experienced more severe cocaine and alcohol dependence, psychiatric disorders, and legal and family problems at the 12-month followup. Feingold and colleagues (in press) found that type A alcohol abusers and cocaine abusers reported less use of their drug of choice at a 6-month followup than did type B subjects, but such differences did not occur among marijuana or opiate abusers.

Alcoholism typologies may be especially useful in treatment-matching studies, which attempt to determine which types of treatments work best for which types of patients. Litt and colleagues (1992) randomly assigned subjects from a sample of 79 alcoholic men to either of two treatment types. Interactional group therapy emphasizes the importance of functioning in relationships, whereas coping-skills training provides basic instruction in coping with both relapse and situations that increase the risk of relapse. Type A alcoholics did better in interactional group therapy than in coping-skills training, whereas the reverse was true for type B subjects. These differences were maintained for 2 years following the beginning of aftercare treatment.

## Which Are the Most Important Dimensions?

Because of its complexity, the clinical application of a multidimensional typological assessment may be impractical as it is currently defined (i.e., as multiple dimensions) and constructed (i.e., using a statistical technique). Such a typology is unlikely to gain wide clinical acceptance (e.g., for guiding treatment decisions) unless it can be implemented quickly and easily by clinicians with a range of expertise. Consequently, in several studies reviewed here, the researchers attempted to identify the more important dimensions by statistically predicting subjects’ classification types based on the subjects’ scores on a subset of these dimensions (table 3).

Ball and colleagues (1995) found that across the three subject samples (i.e., inpatient, outpatient, and community members), antisocial personality and alcohol-dependence severity were the most effective single dimensions predicting cocaine abuse types. Other dimensions found to be important in more than one study include current and lifetime dependence severity, childhood behavior problems, increased AOD usage to avoid withdrawal symptoms, and AOD-related medical problems. One of the most commonly used typology dimensions for AOD abusers—family history—did not emerge by itself as an important variable in these studies.

## Conclusions

Selecting the appropriate typology dimensions for categorizing a population does not depend solely on the ability of the dimensions to cluster subjects statistically. One must first decide the purpose of the assessment. The dimensions that are best for statistically grouping subjects may differ from the dimensions that are most important for understanding the cause and course of the disorder. For example, certain genetically influenced vulnerability factors (e.g., family history, childhood temperament, and behavior problems) may predispose subjects to a more severe form of AOD abuse with worse outcome (i.e., type B). This suggests that one could identify higher risk type B subjects before their problems become severe. Subjects lacking these risk factors (i.e., type A subjects) may develop a less severe and more treatable form of AOD abuse that is more environmentally influenced.

Similarly, the dimensions that are most important for identifying high-risk subjects may differ greatly from the dimensions that are most important for selecting specific treatments once a disorder has become severe. A simpler typology may be useful for some purposes (e.g., patient placement), whereas a more complex model may be better for other purposes (e.g., guiding theory and research). An important research area will be determining which dimensions are of greater significance in defining a general typological system for all AOD abusers. This may be a complicated task, because the relative importance of specific dimensions may vary depending on gender, culture, and the setting and purpose of assessment. Thus, the development of an assessment measure to classify multidimensional types for clinical and prevention purposes is also an important area for research.

Given some of the variability in findings reviewed here, it seems premature to eliminate any typology dimensions from consideration. One could even argue that the A and B typology is too narrow, because it does not include important biological dimensions, such as neurotransmitter systems or physiological reactivity.^6^ Single dimensions may serve specific purposes or patient subgroups. When multiple dimensions are considered together, however, the type A-type B distinction seems to have broad clinical applicability across a range of people and situations.

## Figures and Tables

**Table 1:** Comparison of Single–Dimension With Multidimensional Typology Systems for Alcoholism

Single-Dimension Typologies		
**Etiology** Substance abuse, antisocial personality, depression/anxiety ❑ Family history positive ❑ Family history negative **Gender** ❑ Male ❑ Female **Personality**^1^ ❑ Neurotic ❑ Psychotic ❑ Psychopathic deviate **Age of Onset** ❑ Early (before age 18 or 21) ❑ Later (after age 18 or 21) **AOD**^2^ **Use** ❑ Low severity ❑ High severity **Psychopathology** ❑ Low vs. high psychiatric severity ❑ Depression/anxiety ❑ Antisocial personality disorder		

**Multidimensional Typologies**		

**Dimensions**	**Type A/Type I**	**Type B/Type II**

**Etiology**	More environmental	More genetic
**Gender**	Equal number of males and females	More males
**Personality**^1^	Low impulsivity and novelty seeking	High impulsivity, novelty seeking
**Childhood**	Fewer early risk factors	Conduct disorder
**Age of Onset**	Later	Earlier
**AOD Use**	Less severe, more episodic	More chronic and severe, polydrug use
**Psychopathology**	Lower severity	Higher severity, more antisocial

1 Personality factors are based on the Minnesota Multiphasic Personality Inventory.

2 AOD = Alcohol and other drugs.

**Table 2:** Typology Dimensions Used in Cluster Analysis Studies of Alcohol^1^,^2^ and Cocaine^3^ Users

Alcohol Typology Dimensions	Cocaine Typology Dimensions
**Premorbid Risk Factors**	**Premorbid Risk Factors**
Familial alcoholism	Familial AOD^4^ use
Childhood disorders	Childhood disorders
Impulsiveness/reward seeking	Sensation seeking
Age of onset of problem drinking	Age of onset of drug abuse
**AOD Use: Chronicity and Consequences**	**AOD Abuse**
Frequency of alcohol use (ounces per day)	Frequency of cocaine use (days per month)
Years of heavy drinking	Years of heavy cocaine use
Lifetime severity	Recent AOD-use severity
Alcohol-dependence syndrome	Cocaine-dependence syndrome
Benzodiazepine use	Alcohol-dependence syndrome
Polydrug use	Polydrug use
Physical condition/physical consequence	Severity of medical problems/need for medical treatment
Social consequences	Psychosocial impairment
Relief drinking	
**Psychiatric Symptoms**	**Psychiatric Problems**
Depression	Depression symptoms
Antisocial personality	Antisocial personality
Anxiety	Psychiatric severity
	Lifetime psychiatric diagnoses

1 Babor et al. 1992*a*.

2 Babor et al. 1992*b*.

3 Ball et al. 1995.

4 AOD = Alcohol and other drugs.

**Table 3:** Specific Typology Dimensions That Predicted Type A Versus Type B in Different Studies

Typology Dimensions Used in Various AOD^1^-Use Studies			

Brown et al. (1994) (Alcohol Use)	Schuckit et al. (1995) (Alcohol Use)	Ball et al. (1995) (Cocaine Use)	Feingold et al. (in press) (AOD Use)
Child symptoms	Harm avoidance	Child symptoms	
Lifetime severity	Lifetime severity	Years of use	Lifetime severity
Medical problems	Medical problems	Psychosocial impairment	Psychiatric severity
Dependence severity	Dependence severity	Dependence severity	
Antisocial personality	Relief drinking	Antisocial personality	Withdrawal avoidance
		Age of onset	

1 AOD = Alcohol and other drugs.

## Footnotes

1 AOD-use disorders include both abuse and dependence. These two terms overlap in the alcoholism literature. In general, abuse implies harmful consequences without addiction, whereas dependence refers to addiction (e.g., alcoholism). Unless otherwise noted, this article uses the term “abuse” to include both abuse and dependence in accordance with the terminology used in most of the references cited.

2 Although the Babor, Cloninger, and Schuckit typologies are discussed together because of their similarities, they are not actually equivalent in detail.

3 ASPD is characterized by a pattern of antisocial and irresponsible behavior.

4 First-degree relatives include parents, offspring, and siblings.

5 The remaining two types are defined largely on the basis of alcoholism course and psychological development factors and are slightly more evident in men.
